# The KRAS/Lin28B axis maintains stemness of pancreatic cancer cells via the let‐7i/TET3 pathway

**DOI:** 10.1002/1878-0261.12836

**Published:** 2020-11-28

**Authors:** Yawen Liu, Dawei Wang, Meng Zhou, Hui Chen, Huizhi Wang, Jingyu Min, Jiaxi Chen, Shuhui Wu, Xiufan Ni, Youli Zhang, Aihua Gong, Min Xu

**Affiliations:** ^1^ Department of Gastroenterology Affiliated Hospital of Jiangsu University Jiangsu University Zhenjiang China; ^2^ Department of Cell Biology School of Medicine Jiangsu University Zhenjiang China

**Keywords:** KRAS, let‐7, Lin28B, pancreatic cancer, TET3

## Abstract

Increasing evidence demonstrates that Lin28B plays critical roles in numerous biological processes including cell proliferation and stemness maintenance. However, the molecular mechanisms underlying Lin28B nuclear translocation remain poorly understood. Here, we found for the first time that KRAS promoted Lin28B nuclear translocation through PKCβ, which directly bound to and phosphorylated Lin28B at S243. Firstly, we observed that Lin28B was upregulated in pancreatic cancer, contributing to cellular migration and proliferation. Furthermore, nuclear Lin28B upregulated TET3 messenger RNA and protein levels by blocking the production of mature let‐7i. Subsequently, increased TET3 expression could also promote the expression of Lin28B, thereby forming a Lin28B/let‐7i/TET3 feedback loop. Our results suggest that the KRAS/Lin28B axis drives the let‐7i/TET3 pathway to maintain the stemness of pancreatic cancer cells. These findings illuminate the distinct mechanism of Lin28B nuclear translocation and its important roles in KRAS‐driven pancreatic cancer, and have important implications for development of novel therapeutic strategies for this cancer.

## Graphical abstract

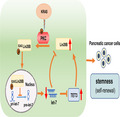

**Abbreviations**
CCK‐8: cell counting kit‐8
CSC: cancer stem cells
IP: immunoprecipitation
MUT: mutant type
NLS: nuclear localization signal
PC: pancreatic cancer
PCSC: pancreatic cancer stem cells
PKC: protein kinase C
WT: wild‐type

## 1 Introduction

Pancreatic cancer (PC) is one of the most common malignant tumors with a dismal 5‐year survival rate [1]. Emerging evidence has shown that cancer stem cells (CSC) play important roles in tumorigenesis, tumor recurrences, metastasis and resistance to the current chemotherapy and radiation [2, 3]. Thus, an explicit understanding of the molecular mechanism in the growth of pancreatic cancer stem cells (PCSC) can provide a new strategy for the clinical treatment of pancreatic cancer. Notably, it has been reported that oncogenic KRAS mutated in approximately 90% of PC have established key roles in the self‐renewal of PCSC to drive pancreatic neoplasia [4, 5, 6, 7]. However, its mechanism for maintaining the stemness and self‐renewal of PCSC needs to be further clarified.

The RNA‐binding protein Lin28, which was first identified in the nematode *Caenorhabditis elegans*, has an essential function in advanced human malignancies including pancreatic cancer [8, 9]. Lin28 could reprogram human fibroblasts into pluripotent stem cells, along with OCT4, SOX2 and NANOG, further confirming its role in the regulation and maintenance of CSC [10, 11]. It is found that Lin28 could selectively block the expression of let‐7 microRNAs [12]. Functionally, Lin28A recruits a terminal uridylyltransferase (TUTase), Zcchc11, to block pre‐let‐7 processing by Dicer in the cytoplasm, while nuclear Lin28B directly binds and sequesters pri‐let‐7 away from the Microprocessor, which represses the expression of let‐7 [13]. Thereby the decrease of let‐7 blocked by Lin28 plays critical roles in CSC maintenance by increasing let‐7 miRNA targets, such as KRAS, c‐Myc, BLIMP1 and HMGA [14, 15]. However, the molecular mechanism of Lin28B nuclear translocation and its role in the development and progression of pancreatic cancer remained unclear.

In this study, we found that Lin28B was significantly more highly expressed in pancreatic cancer tissues than in the pancreatic cancer adjacent tissues, and showed that KRAS promoted the nuclear translocation of Lin28B by protein kinase C (PKCβ). Moreover, nuclear Lin28B increased TET3 expression by repressing mature let‐7i; increased TET3 would feed back to promote the expression of Lin28B, which maintains the stemness of PC cells. Therefore, our results illuminate the distinct mechanism of Lin28B nuclear translocation and suggest that the KRAS/Lin28B axis drives the let‐7i/TET3 pathway to maintain the stemness of pancreatic cancer cells.

## 2 Materials and methods

### 2.1 Clinical samples

All clinical pancreatic cancer specimens and matched adjacent non‐tumor tissues were obtained from newly diagnosed patients undergoing surgery at Changhai Hospital, Shanghai. All specimens were immediately frozen in liquid nitrogen for further analysis.

Before collecting samples, a written informed consent form was collected from each patient. The present study conformed to the standards set by the Declaration of Helsinki and approved by the Ethics Committee of Jiangsu University Affiliated Hospital.

### 2.2 Cells and cell culture

The human pancreatic cancer cell lines PANC1, SW1990 and PaTu8988 cells were bought from Shanghai Institutes for Biological Sciences (CAS) and the human embryonic kidney cell line (HEK293T) was obtained from the American Type Culture Collection (ATCC, Manassas, VA, USA). All cells were tested and authenticated by short tandem repeat DNA typing analysis. SW1990 cells were cultured in L‐15 (HyClone, Beijing, China); PANC1, PaTu8988 and HEK293T cells were cultured in DMEM (HyClone). All media were supplemented with 10% fetal bovine serum (Gibco, Carlsbad, CA, USA). Cells were cultured in a humidified atmosphere with 5% CO_2_ at 37 °C.

### 2.3 Plasmid construction

The entire Lin28B sequence was amplified with RT‐PCR using primers Lin28B‐all‐F, 5′‐CGGGGTACCAATGGCCGAAGGCGGGGCTA‐3′ and Lin28B‐all‐R, 5′‐CGCGGATCCTTATGTCTTTTTCCTTTTT‐3′ and then cloned into the expression vector p3xFLAG‐Myc‐CMV™‐24 (Sigma‐Aldrich, San Francisco, CA, USA). The Lin28B, TET3, PKCβ and KRAS short hairpin (sh)RNA oligos (Lin28B‐shRNA‐F, 5′‐CCGGGCAGGCATAATAAGCAAGTTACTCGAGTAACTTGCTTATTATGCCTGCTTTTTG‐3′; Lin28B‐shRNA‐R, 5′‐AATTCAAAAAGCAGGCATAATAAGCAAGTTACTCGAGTAACTTGCTTATTATGCCTGC‐3′; TET3‐shRNA‐F, 5′‐CCGGGAACCTTCTCTTGCGCTATTTCTCGAGAAATAGCGCAAGAGAAGGTTCTTTTTG‐3′; TET3‐shRNA‐R, 5′‐AATTCAAAAAGAACCTTCTCTTGCGCTATTTCTCGAGAAATAGCGCAAGAGAAGGTTC‐3′; sh‐PKCβ‐F, 5′‐CCGGCAAGTTTAAGATCCACACGTACTCGAGTACGTGTGGATCTTAAACTTGTTTTT‐3′; sh‐PKCβ‐R, 5′‐AATTCAAAAACAAGTTTAAGATCCACACGTACTCGAGTACGTGTGGATCTTAAACTTG‐3′; sh‐KRAS‐F, 5′‐CCGGCTATACATTAGTCCGAGAAATCTCGAGATTTCTCGGACTAATGTATAGTTTTTG‐3′; sh‐KRAS‐R, 5′‐AATTCAAAAACTATACATTAGTCCGAGAAATCTCGAGATTTCTCGGACTAATGTATAG‐3′) produced by Sangon Biotech Co. Ltd. (Shanghai, China), were first annealed into double strands and then cloned into pLKO.1‐puro or Tet‐on‐pLKO‐puro vector.

### 2.4 Cell transfection and generation of stable cell lines

According to the manufacturer’s instructions, cells were transfected with plasmid DNA or shRNA using Lipofectamine 2000 reagent (Invitrogen, Carlsbad, CA, USA). Using Lipofectamine 2000, sh‐Lin28B, packaging plasmid psPA×2 and envelope plasmid pMD2.G were co‐transfected into HEK293T cells. Viruses were collected 48 and 72 h after transfection, and 1 × 10^6^ recombinant lentiviruses were used to infect the cell transduction unit in the presence of 8 mg·mL^−1^ polyethylene (Sigma‐Aldrich). Cells were selected by puromycin (2 µg·mL^−1^) until all cells in the blank group died.

### 2.5 Western blotting

The cultured cells were washed with cold PBS and then treated with cell lysis buffer (2× loading buffer, 2 μg·mL^−1^ Aprotinin, 1 mm PMSF, 2 mm β‐mercaptoethanol) at 100 °C for 10 min. Then the mixture was centrifuged under 4 °C at 14000***g*** for 10 min. Approximately 10 μL of protein was loaded in each lane of 10% SDS/PAGE gel and separated, and the protein then transferred to a PVDF membrane. The membrane was blocked with 5% BSA for 1 h at room temperature and then incubated with primary antibodies at 4 °C overnight, followed by the secondary antibody. The antibodies used were rabbit anti‐CD133 (Cell Signaling Technology, Danvers, MA, USA; #64326), rabbit anti‐OCT4 (Cell Signaling Technology; #2750), rabbit anti‐SOX2 (Cell Signaling Technology; #3579), rabbit anti‐NANOG (Cell Signaling Technology; #4903), mouse anti‐KRAS (Santa Cruz Biotechnology, Dallas, TX, USA; sc30), mouse anti‐β‐Tubulin (Cell Signaling Technology; #6181), rabbit anti‐TET3 (Cell Signaling Technology; #85016), rabbit anti‐Lin28B (Signalway Antibody LLC, College Park, MD, USA；#21626), rabbit anti‐PKCβ (Cell Signaling Technology; #46809), rabbit anti‐PKCδ (Cell Signaling Technology; #2058), rabbit anti‐PKCζ (Cell Signaling Technology; #9372), rabbit anti‐PKCμ (Cell Signaling Technology; #2056) and mouse anti‐Flag (Sigma; CAT F1804).

### 2.6 Real‐time PCR

According to the manufacturer’s protocol, total RNA was isolated using RNAiso Plus (Takara, Dalian, China). RevertAid First Strand cDNA Synthesis Kit (Thermo Fisher Scientific, Waltham, MA, USA) was used for reverse transcription according to the manufacturer’s recommendations. SYBR green‐based real‐time PCR was then performed in triplicate, and GAPDH was used as an internal control. Primers for the qRT‐PCR were as follows: GAPDH primer: forward, 5′‐GGTGAAGGTCGGTGTGAACG‐3′ and reverse, 5′‐CTCGCTCCTGGAAGATGGTG‐3′; Lin28B primer forward, 5′‐CAGCCAAAG AAGTGCCATTA‐3′ and reverse, 5′‐TTCTGCTTCCTGTCTTCCCT‐3′; TET3 primer forward, 5′‐GTTCCTGGAGCATGTACTTC‐3′ and reverse, 5′‐CTTCCTCTTT GGGATTGTCC‐3′. The relative fold change in RNA expression was calculated using the 2^−ΔΔCT^ method.

### 2.7 Cell counting kit‐8 proliferation assay

Cell proliferation efficiency was assessed by using a Cell counting kit‐8 (CCK‐8). Cells (2 × 10^3^) were seeded into each well of a 96‐well culture plate and incubated in an incubator at 37 °C for 1, 2, 3, 4 and 5 days. After removal of the existing culture solution, 100 μL serum‐free culture medium and 10 μL CCK8 solutions were added to each well. The absorbance was measured with a plate reader at 450 nm after incubating for 2 h.

### 2.8 Colony formation assay

Stable cell lines (PANC1, SW1990 and PaTu8988 cells) were harvested and seeded into six‐well plates (500 cells per well) and cultured for 2 weeks. Colonies were fixed in 4% paraformaldehyde for 15 min and then stained with 0.05% crystal violet for 30 min. Photomicrographs were taken and the number of colonies per well was counted.

### 2.9 Wound healing assay

Cells were seeded in 24‐well plates and cultured to complete confluence. At time 0, a 10‐μL pipette tip was used to scratch the diameter after the medium was removed. The scraped cells were gently washed away with PBS, and then the cells were cultured with serum‐free medium. The distance was recorded at 0, 12 and 24 h using an inverted microscope.

### 2.10 Invasion assay

Cell invasion assays were performed as per previously published protocols [16].

### 2.11 Immunoprecipitation assay

Cells were lysed in immunoprecipitation (IP) lysis buffer containing protease inhibitors for 30 min at 4 °C. The supernatant was then harvested after centrifuged under 4 °C at 12 000 ***g*** for 10 min. The pre‐cleared supernatant was immunoprecipitated with 1 μL anti‐Flag antibody and 25 μL of Protein A/G beads. After washing with IP buffer, the protein complexes were collected. The input and immunocomplexes were analyzed by Western blotting.

### 2.12 Preparation of nuclear and cytoplasmic protein extracts

The nuclear and cytoplasmic protein were extracted as per previously published protocols [17].

### 2.13 Immunofluorescence assay

For immunofluorescence, cells were grown on coverslips in a 24‐well plate for 48 h and fixed with ice‐cold 3% paraformaldehyde for 15 min at room temperature after washing twice with PBS, and then blocked with 3% BSA for 1 h. Cells were incubated with primary antibody at 4 °C overnight. After being rewarmed for 1 h and washed three times with PBS, the cells were incubated with specific secondary antibodies for 2 h at 37 °C in the dark. After washing three times with PBS, the nuclei were stained with DAPI (1 μg·mL^−1^; Pierce, Rockford, IL, USA) for 5 min at room temperature. The fluorescence images were captured with a confocal microscope (DeltaVision Elite; GE Healthcare, Waukesha, WI, USA).

### 2.14 Luciferase reporter assay

HEK293T cells were co‐transfected with the indicated luciferase reporter and let‐7i or microRNA (miRNA) negative control. Forty‐eight hours after transfection, luciferase activity was detected by a dual‐luciferase reporter assay system according to the manufacturer’s instructions. Results represented the average of triplicate samples from three independent experiments.

### 2.15 Motif scan and TargetScan analysis

To gain further insight into the molecular mechanism of Lin28B in PC progression, we analyzed the sequence of Lin28B to predict the functional motif through GPS2.1 software and Motif scan (http://scansite.mit.edu). Using the Motif scan, we found that Lin28B interacted with PKC at two conserved sequences (Δ112–125 and Δ239–249), which were the nuclear localization signal (NLS) of Lin28B (Fig. 2A). TargetScan (http://www.targetscan.org/mamm_31/) was used to search for potential miRNA that had a chance to interact with Lin28B and TET3. We found that let‐7i has a potentialL, DWW, MZ, HC, HZW, JYM, JXC, SHW and XFN carried out the complementary binding site with the binding site in the 3′ UTR of Lin28B and TET3 (Fig. 5A).

### 2.16 Statistical analysis

All data are presented as mean ± SD from at least three independent experiments. Comparisons between groups were analyzed using Student’s *t*‐test (two groups) or one‐way ANOVA (multiple groups) using graphpad prism 5 software (San Diego, CA, USA). *P* < 0.05 was considered statistically significant.

## 3 Results

### 3.1 Lin28B enhances the abilities of migration and proliferation in pancreatic cancer cells

**Fig. 1:**
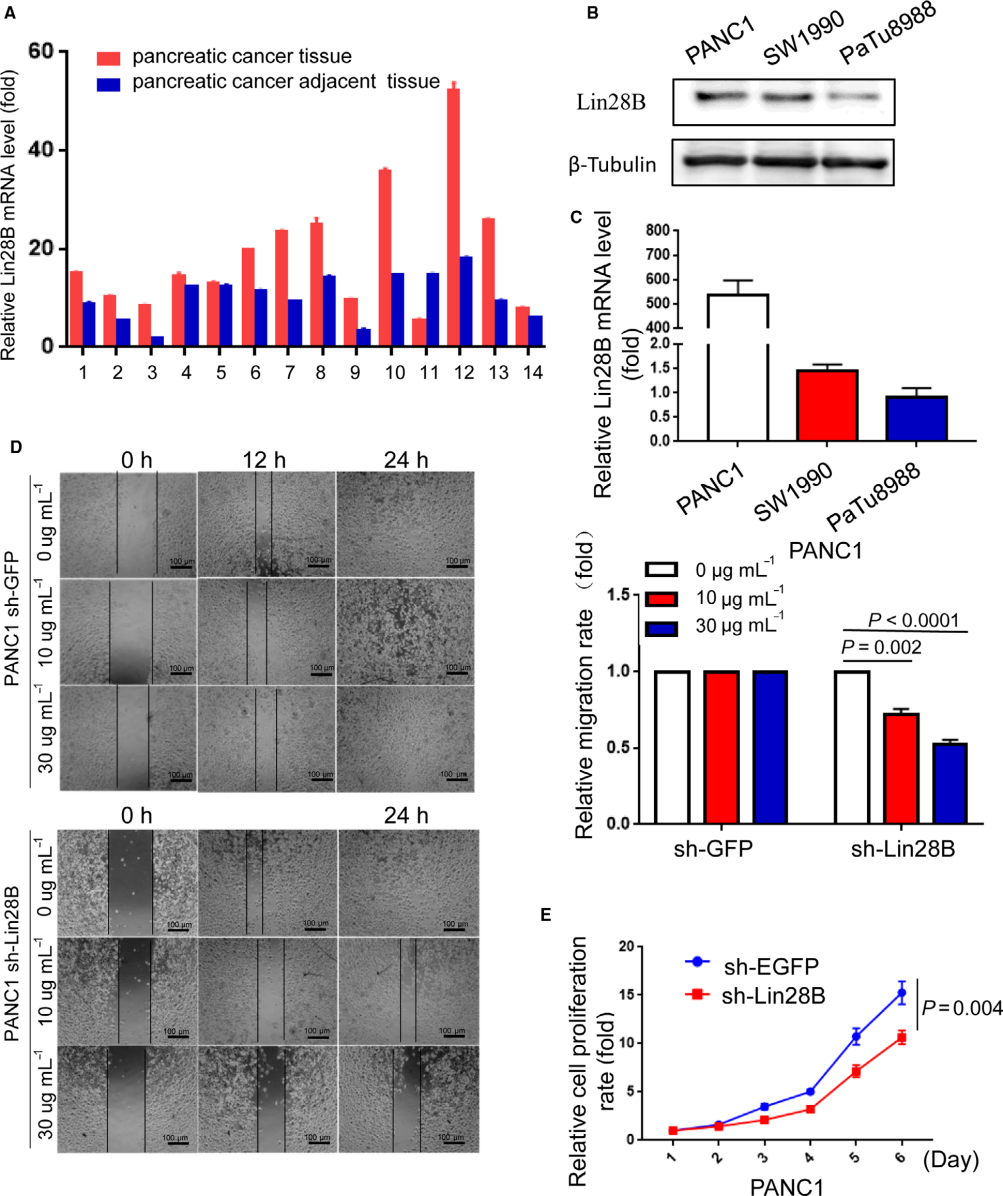
Lin28B enhances migration and proliferation in pancreatic cancer cells. (A) Lin28B expression is upregulated in PC tissues compared with that in normal pancreatic tissues. (B,C) The relative expression of Lin28B protein and mRNA in different PC cell lines (PANC1, SW1990 and PaTu8988) was assessed by Western blotting and qRT‐PCR, respectively. (D) The ability of invasion was examined by using wound‐healing assay in PANC1 transfected with sh‐GFP or sh‐Lin28B plasmids. The histogram represents the statistical analysis of the wound‐healing assays. (E) The effect of Lin28B on cell growth was assessed by a CCK‐8 assay in PANC1 with Lin28B knockdown. All experiments were repeated three times. Each error bar represents the standard error of the mean (SEM). Statistical analysis was performed using the unpaired Student’s *t*‐test or one‐way ANOVA test.

To determine whether Lin28B is involved in pancreatic cancer progression, we first analyzed Lin28B messenger RNA (mRNA) levels in 14 pairs of PC tissues and paired adjacent normal tissues. Compared with the adjacent normal tissues, Lin28B expression was significantly higher in pancreatic cancer tissues (Fig. 1A). Furthermore, we investigated the relative expression of Lin28B protein and mRNA in different PC cell lines (PANC1, SW1990 and PaTu8988) using Western blotting and qRT‐PCR. The data showed that Lin28B expression, at both mRNA and protein levels, was higher in PANC1 cells than that in SW1990 and PaTu8988 cells, but relatively lower in PaTu8988 cells (Fig. 1B,C). To further determine the role of Lin28B in PC, we examined the effect of Lin28B on pancreatic cancer cell migration by wound healing assays. We knocked down Lin28B in PANC1 cells after doxycycline (dox) induction, and found that Lin28B downregulation largely suppressed the migratory ability of PANC1 cells (Fig. 1D). Meanwhile, we performed CCK‐8 assay to examine the cellular proliferation. We found that Lin28B downregulation largely suppressed the proliferative activity of PANC1 cells (Fig. 1E). These results revealed that Lin28B was significantly enriched in pancreatic cancer tissues and promoted the migration and proliferation of pancreatic cancer cells.

### 3.2 PKCβ promotes Lin28B nuclear translocation

**Fig. 2:**
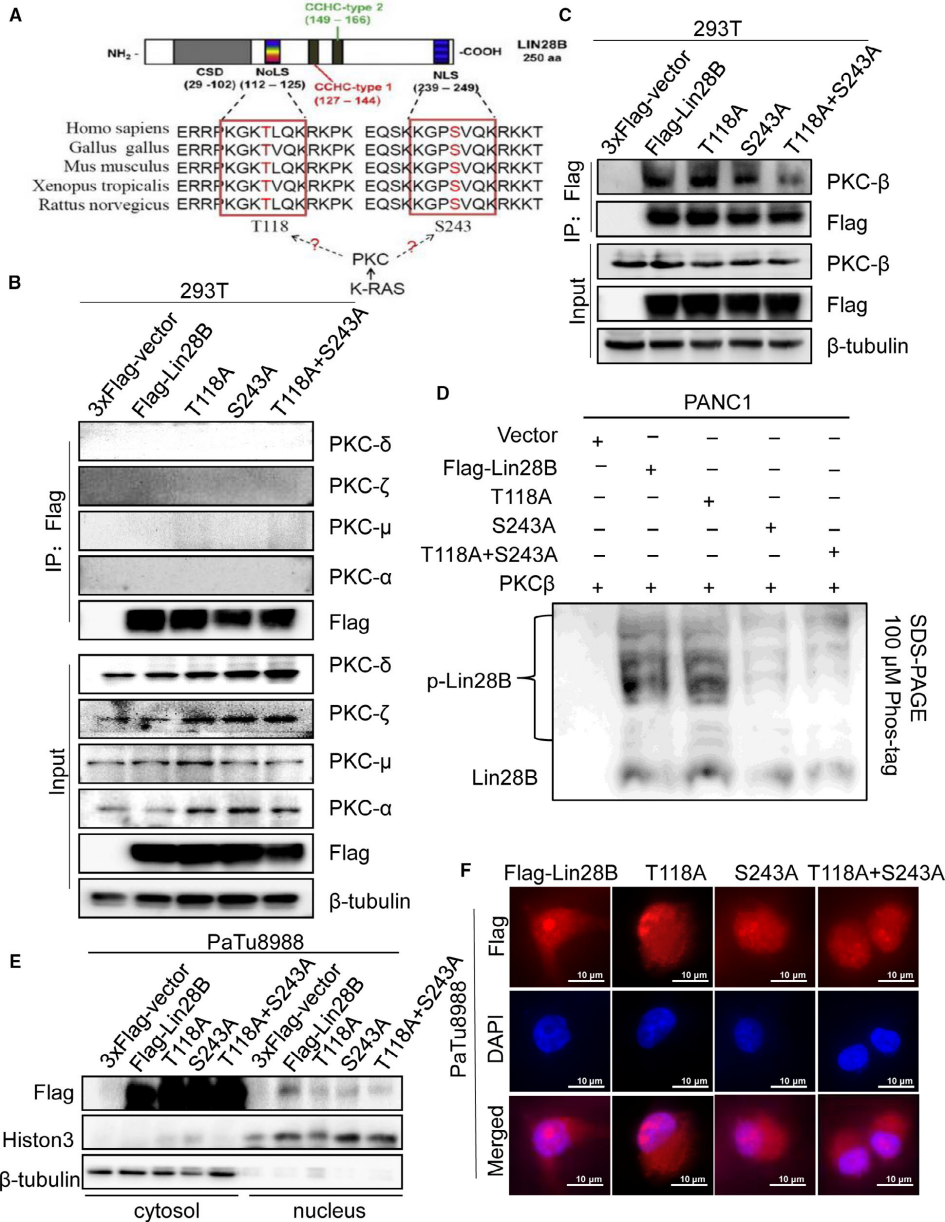
PKCβ promotes Lin28B nuclear translocation. (A) The bioinformatics prediction of the PKC binding sequence in Lin28B is shown. NLS, nuclear localization signal; NoLS, nucleolar localization signal. (B,C) To identify PKC could interact with Lin28B, HEK293T cells were transfected with either wild‐type (WT), or mutation (T118A or S243A) Flag‐tagged Lin28B and immunoprecipitated with anti‐Flag antibody. (D) Samples separated by conventional SDS/PAGE were loaded on the Phos‐tag SDS/PAGE gel and assayed by immunoblotting using an anti‐Lin28B antibody. p‐Lin28B, phosphorylated Lin28B. (E, F) Lin28B nuclear localization after Flag‐tagged Lin28B WT, T118A, S243A, or T118A and S243A treatment were analyzed by nuclear‐plasmin extraction and immunofluorescence, respectively. All experiments were repeated three times.

To identify the molecular mechanism whereby Lin28B regulates PC cells, we further analyzed the sequence of Lin28B to predict the functional motif through GPS2.1 software and Motif scanner (http://scansite.mit.edu/motifscan_seq.phtml). We found that Lin28B interacted with PKC at two conserved sequences (Δ112–125 and Δ239–249), which were NLS of Lin28B [13] (Fig. 2A). PKC represents a family of related isoforms involved in cellular proliferation, differentiation and survival [18]. To determine which member of PKC can interact with Lin28B and whether PKC can bind to Lin28B at the predicted conserved sequences, we further performed IP assays using an anti‐Flag antibody, and T118 or S243 mutated to alanine (A), respectively. As shown in Fig. 2B,C, Lin28B could interact with PKCβ, and the interaction was reduced when S243 was mutated, but not T118, which indicated that S243 was critical to Lin28B–PKCβ binding. We also collected samples of PANC1 cells transfected with Flag‐Lin28B, T118A, S243A or double mutant Flag‐Lin28B (T118A and S243A), and performed IP analysis with the anti‐Flag antibody. These samples were then put on the Phos‐tag SDS/PAGE gel to examine the level of Lin28B phosphorylation. We observed that PKCβ could phosphorylate Lin28B in Flag‐Lin28B and T118A groups, whereas the level of phospho‐Lin28B was reduced in S243A and double mutant Flag‐Lin28B (T118A and S243A) groups (Fig. 2D). It is suggested that PKCβ could directly phosphorylate S243.

To test the function of these NLS, we performed nuclear/cytoplasmic extraction and immunofluorescence. Western blotting showed nuclear Lin28B decreased after S243A treatment, whereas the double mutant Flag‐Lin28B (T118A and S243A) showed a more significant decrease in nuclear localization (Figs 2E and S1B). Immunofluorescence analysis showed that S243A transfected cells resulted in more decreased Lin28B nuclear localization compared with the T118A group (Figs 2F and S1C).

### 3.3 KRAS regulates the nuclear translocation of Lin28B through PKCβ

**Fig. 3:**
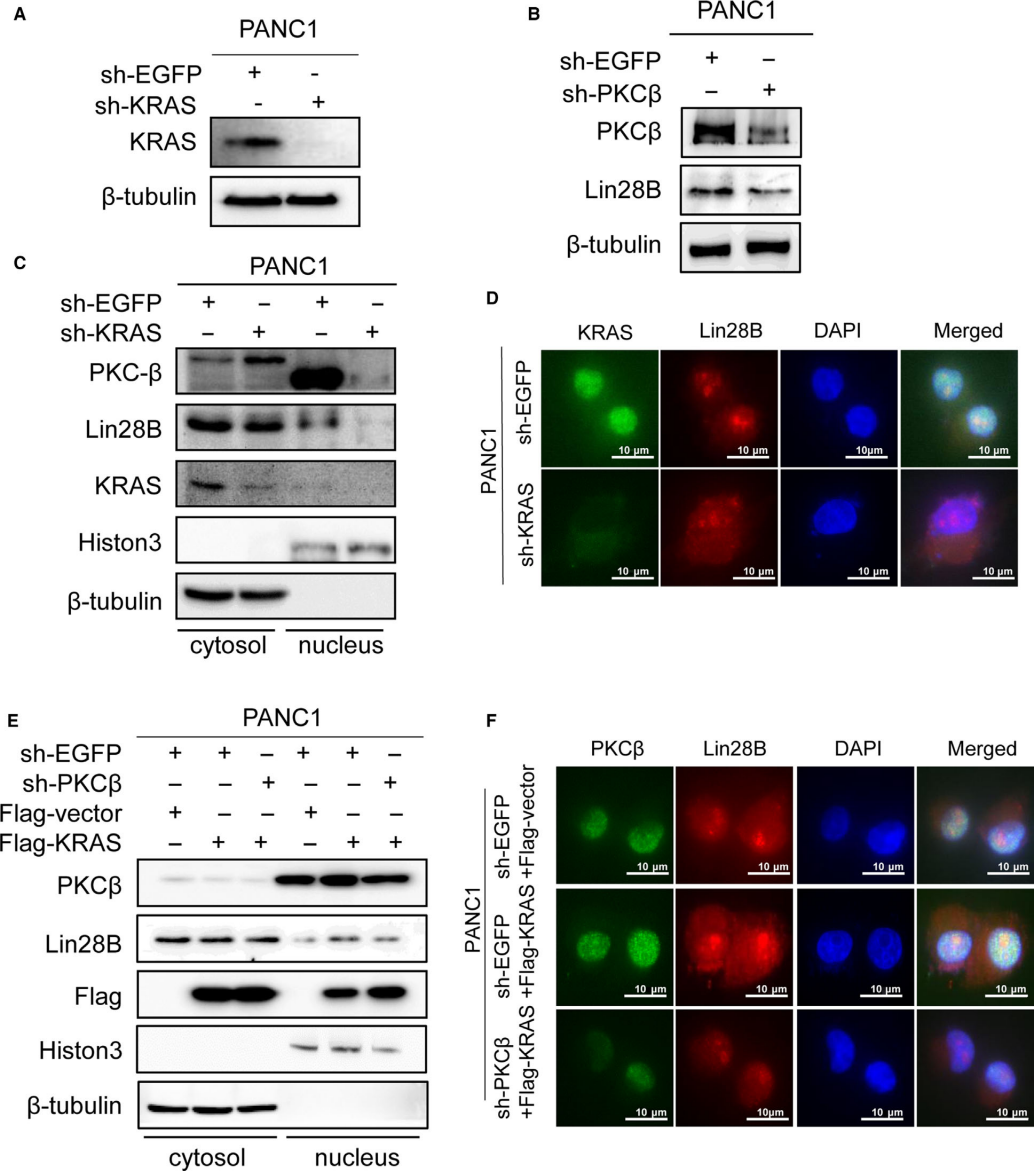
KRAS regulates nuclear translocation of Lin28B through PKCβ. (A) The expression of KRAS was assessed by Western blotting in PANC1 cells after transfection with sh‐KRAS. (B) PKCβ and Lin28B protein levels were reduced in PANC1 cells after sh‐PKCβ treatment. (C) Western blotting analysis of the nuclear/cytoplasmic fraction revealed the subcellular localization of Lin28B in PANC1 after transfection with sh‐KRAS. (D) Immunofluorescence showed Lin28B nuclear localization in PANC1 transfected with sh‐KRAS. (E) Lin28B nuclear localization was analyzed by transfection with Vector, Flag‐KRAS or Flag‐KRAS and sh‐PKCβ. (F) The distribution of Lin28B in PANC1 with KRAS overexpression and PKCβ knockdown was studied by immunofluorescence. All experiments were repeated three times.

Oncogenic mutations of KRAS give rise to a GTP‐bound protein that constitutively activates the effectors to promote initiation, progression and metastasis of PC [19]. KRAS oncogenic activity could be modulated by several reversible post‐translational modifications of KRAS, and phosphorylation at Ser‐181 of oncogenic KRAS is required for survival and tumorigenic activity [20, 21]. In addition, KRAS facilitates sustained activation status for PKC, and PKC in turn activates KRAS by phosphorylation at Ser‐181 [22, 23]. Having demonstrated that Lin28B was phosphorylated by PKC and translocated from the cytosol to the nucleus, we speculated that KRAS might also be involved in the process of Lin28B nuclear translocation through PKCβ. Subsequently, we constructed sh‐KRAS and sh‐PKCβ plasmids to investigate the role of KRAS in Lin28B nuclear translocation, using sh‐EGFP as a control. The expression of KRAS was decreased in the sh‐KRAS group compared with the sh‐EGFP group (Fig. 3A). PKCβ knockdown was also examined in PANC‐1 cells, and the protein levels of PKCβ and Lin28B were significantly downregulated in the sh‐PKCβ group compared with the sh‐EGFP group (Fig. 3B).

To determine the subcellular localization of Lin28B in the PC cells, we performed nuclear/cytoplasmic extraction and immunofluorescence. KRAS knockdown resulted in a decrease of nuclear Lin28B and PKCβ, as observed in the Western blotting analysis of the nuclear/cytoplasmic fraction (Figs 3C and S1D). In addition, we validated a decreased nuclear Lin28B in PANC1 cells with KRAS downregulation by immunofluorescence microscopy (Figs 3D and S1E). Most importantly, KRAS upregulation increased the nuclear localization of Lin28B and PKCβ, but we observed a reduction in nuclear Lin28B in PANC1 cells co‐transfected with Flag‐KRAS and sh‐PKCβ (Figs 3E and S2A). Notably, immunofluorescence analysis showed that KRAS promoted nuclear expression of Lin28B, whereas nuclear Lin28B was reduced in PANC1 cells with KRAS overexpression and PKCβ knockdown (Figs 3F and S2B). These data further confirmed that KRAS impacts the nuclear translocation of Lin28B through PKCβ.

### 3.4 Lin28B upregulates TET3 expression in pancreatic cancer cells

**Fig. 4:**
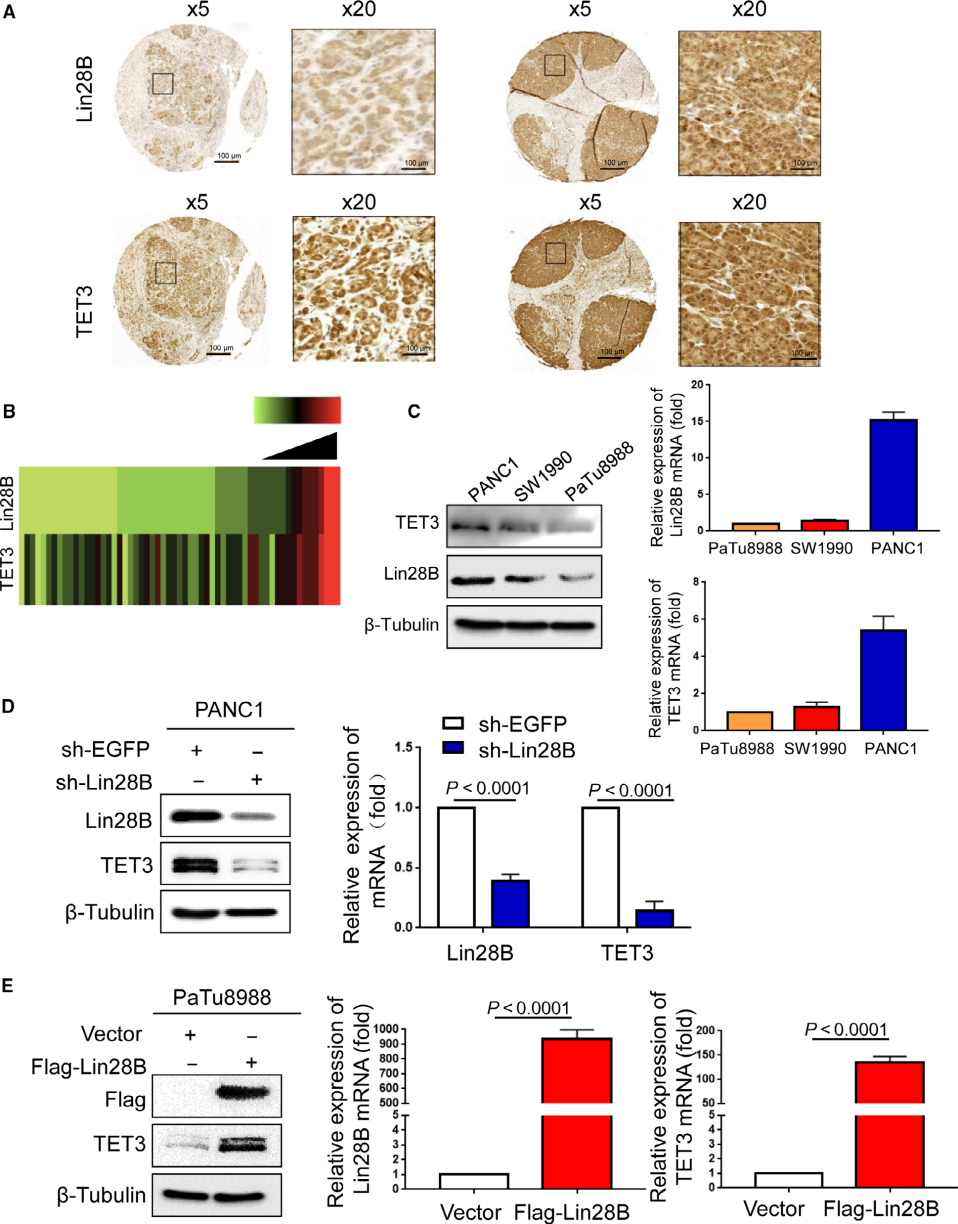
Lin28B enhances TET3 expression in pancreatic cancer cells. (A) Samples from PC patients were prepared for IHC staining with antibodies against Lin28B and TET3. (B) Analysis of the expression of Lin28B correlates with TET3. The results are presented by a heat map. (C) Relative expression protein and mRNA levels of Lin28B and TET3 were assessed in PANC1, SW1990 and PaTu8988 cells. (D) TET3 mRNA and protein levels were detected by real‐time PCR or Western blotting in PANC1 with Lin28B downregulation. (E) Overexpression of Lin28B in PaTu8988 promotes the expression of TET3. All experiments were repeated three times. Each error bar represents the standard error of the mean. Statistical analysis was performed using the unpaired Student’s *t*‐test.

The above results indicate that PKCβ can phosphorylate Lin28B at S243, is regulated by KRAS and translocates Lin28B from the cytosol to the nucleus. However, the mechanism of Lin28B involvement in stemness maintenance of PC cells requires further investigation. Recent studies have shown critical roles of TET3 in the maintenance of PCSC by maintaining hypomethylation levels of pluripotency markers such as OCT4 and NANOG [24, 25]. To further investigate the mechanism of Lin28B involved in stemness maintenance, we first found that Lin28B expression was associated with TET3 in the PC samples analyzed by immunohistochemistry (Fig. 4A,B). Next, we assessed the relative expression of protein and mRNA levels of Lin28B and TET3 in PANC1, SW1990 and PaTu8988 cells. As shown in Fig. 4C, both Lin28B and TET3 protein and mRNA levels were the highest in PANC1 cells, followed by SW1990 cells and the lowest in PaTu8988 cells among three PC cell lines. In addition, we transfected sh‐Lin28B or sh‐EGFP into PANC1 cells to examine TET3 expression.

Compared with the sh‐EGFP group in PANC1 cells, TET3 protein and mRNA levels decreased in sh‐Lin28B cells (Fig. 4D), whereas Lin28B overexpression resulted in significant upregulation of TET3 protein and mRNA levels (Fig. 4E).

### 3.5 Lin28B elevates TET3 expression by repressing let‐7i

**Fig. 5:**
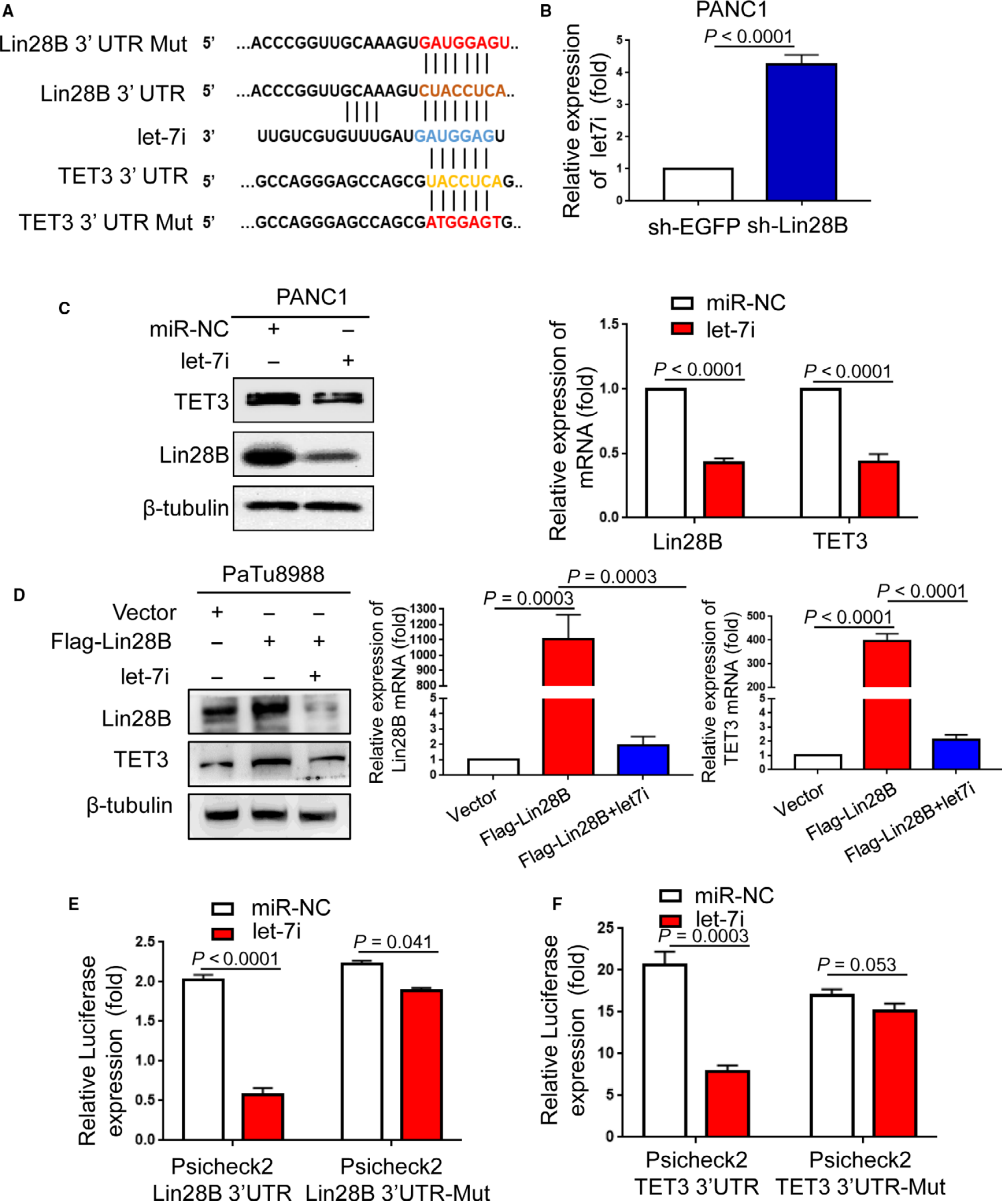
Lin28B elevates TET3 expression by repressing let‐7i. (A) Bioinformatics prediction of let‐7i binding sites in Lin28B 3′UTR or TET3 3′UTR sequence using TargetScan was shown. (B) Lin28B knockdown resulted in significant upregulation of let‐7i in PANC1. (C) The protein and mRNA levels of Lin28B and TET3 were measured in PANC1 transfected with miR‐NC or let‐7i. (D) The protein and mRNA levels of Lin28B and TET3 were assessed in PANC1 transfected with vector, Flag‐Lin28B, or Flag‐Lin28B and let‐7i. (E) The relative luciferase activities were detected by transfecting Lin28B wt or Lin28B mut and miR‐NC or let‐7i into PANC1. (F) Dual‐luciferase assays showed diminished luciferase activity when co‐transfected with TET3 wt, and let‐7i occurred in PANC1. Results represented the average of triplicate samples from three independent experiments. Each error bar represents the standard error of the mean. Statistical analysis was performed using the unpaired Student’s *t*‐test.

To explore the molecular mechanism between Lin28B and TET3, TargetScan, an online miRNA target prediction database, was employed to predict potential miRNA targeting Lin28B and binding to TET3. Using prediction tools, we showed that let‐7i has a potential complementary binding site with the binding site in the 3′ UTR of Lin28B and TET3, as depicted in Fig. 5A. As a highly conserved RNA‐binding protein, Lin28B regulates many biological processes including stem cell differentiation and cell proliferation through blockade of the biogenesis of the let‐7 miRNA family [10, 12, 26]. Let‐7 is frequently downregulated in human cancers, and a reduced expression of let‐7 family members is highly associated with poor prognosis for cancer patients [27, 28]. When we examined mature let‐7i levels in PANC1 cells with Lin28B knockdown, the results showed that Lin28B knockdown resulted in significant upregulation of let‐7i (Fig. 5B). Furthermore, we observed that let‐7i suppressed Lin28B and TET3 protein expression and mRNA levels, as shown in Fig. 5C. To determine whether Lin28B modulates TET3 expression by suppressing let‐7i, we measured TET3 levels in PaTu8988 cells co‐transfected with Flag‐Lin28B and let‐7i. We observed that compared with the control group in PaTu8988 cells, TET3 protein and mRNA levels increased in Flag‐Lin28B cells, whereas the Flag‐Lin28B and let‐7i group did not show a significant response (Fig. 5D).

To clarify that the mechanism of let‐7i action on Lin28B is specific, we performed a luciferase activity assay to detect the potential association between Lin28B and let‐7i. The luciferase reporter assay revealed that the luciferase activity was significantly lower in the wild‐type Lin28B group than in the vector control group, whereas the mutated Lin28B group did not show a significant response to let‐7i, indicating that Lin28B can directly bind to let‐7i (Fig. 5E). Moreover, the bioinformatics analysis and luciferase activity assay showed that let‐7i significantly reduced the luciferase activity of the 3′UTR‐TET3‐WT reporter gene vector, but no significant change was observed for the 3′UTR‐TET3‐MUT vector (Fig. 5F). Taken together, these data suggested that Lin28B modulates TET3 expression by suppressing let‐7i.

### 3.6 Lin28B maintains stemness of pancreatic cancer cells by increasing TET3 expression

**Fig. 6:**
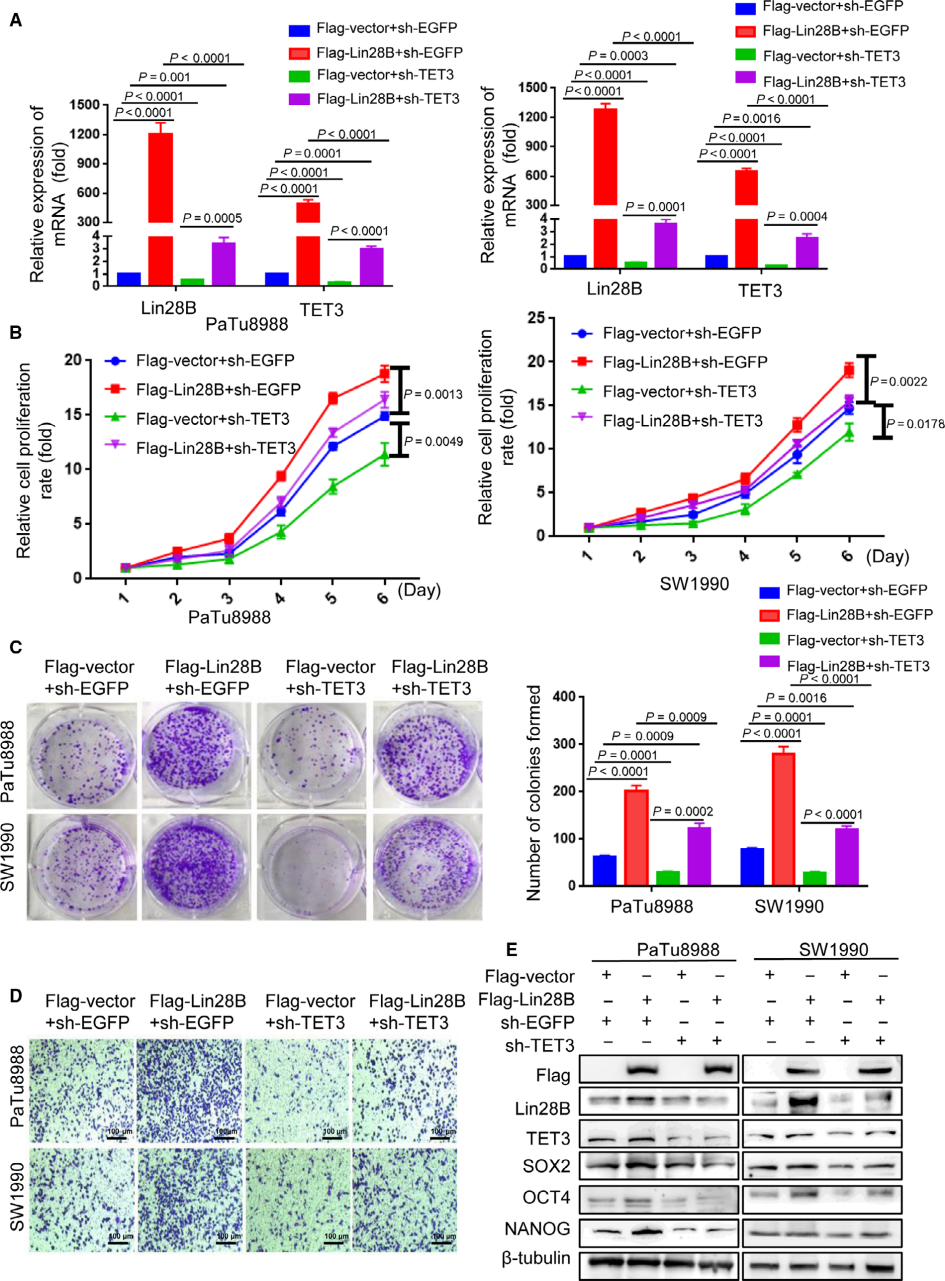
Lin28B maintains the stemness of pancreatic cancer cells via regulation of TET3 expression. (A–E) PaTu8988 and SW1990 cells were co‐transfected Flag‐vector and sh‐EGFP, Flag‐Lin28B and sh‐EGFP, sh‐TET3 and Flag‐vector, or Flag‐Lin28B and sh‐TET3. Relative expression of (A) mRNA levels of Lin28B and TET3, (B) cell proliferation, (C) colony formation capacity, (D) invasive ability and (E) the stem cell markers NANOG, OCT4 and SOX2 were analyzed in the two transfected cells by real‐time PCR, the CCK‐8 assay, the colony formation assay, Transwell assay, or Western blotting analysis, respectively. All experiments were repeated three times. Each error bar represents the standard error of the mean. Statistical analysis was calculated using Student’s *t*‐test or one‐way ANOVA test.

It is known that Lin28B expression is associated with TET3 in PC samples and cell lines, and Lin28B can enhance the expression of TET3 by reducing let‐7i. Therefore, we hypothesized that Lin28B was involved in PC progression through TET3. To test this hypothesis, we co‐transfected Flag‐vector and sh‐EGFP, Flag‐Lin28B and sh‐EGFP, sh‐TET3 and Flag‐vector, or Flag‐Lin28B and sh‐TET3 into PaTu8988 and SW1990 cells. Both the Lin28B and TET3 protein and mRNA expression levels were strongly decreased after knock down of TET3 in PC cells with Lin28B overexpression (Fig. 6A,E). Moreover, the CCK‐8 and colony formation assays revealed that Lin28B upregulation promoted cell proliferation and the number of cell colonies, whereas TET3 knockdown in Lin28B overexpressed cells resulted in suppression of the proliferative capacity (Fig. 6B,C). A Transwell assay was used for the invasion test; the results demonstrated that Lin28B overexpression increased the invasive ability, whereas TET3 knockdown decreased the invasive ability in PC cells with Lin28B overexpression (Fig. 6D). The expression of the stemness markers OCT4, NANOG and SOX2 was reduced when the cells were co‐transfected with Flag‐Lin28B and sh‐TET3 (Fig. 6E).

**Fig. 7:**
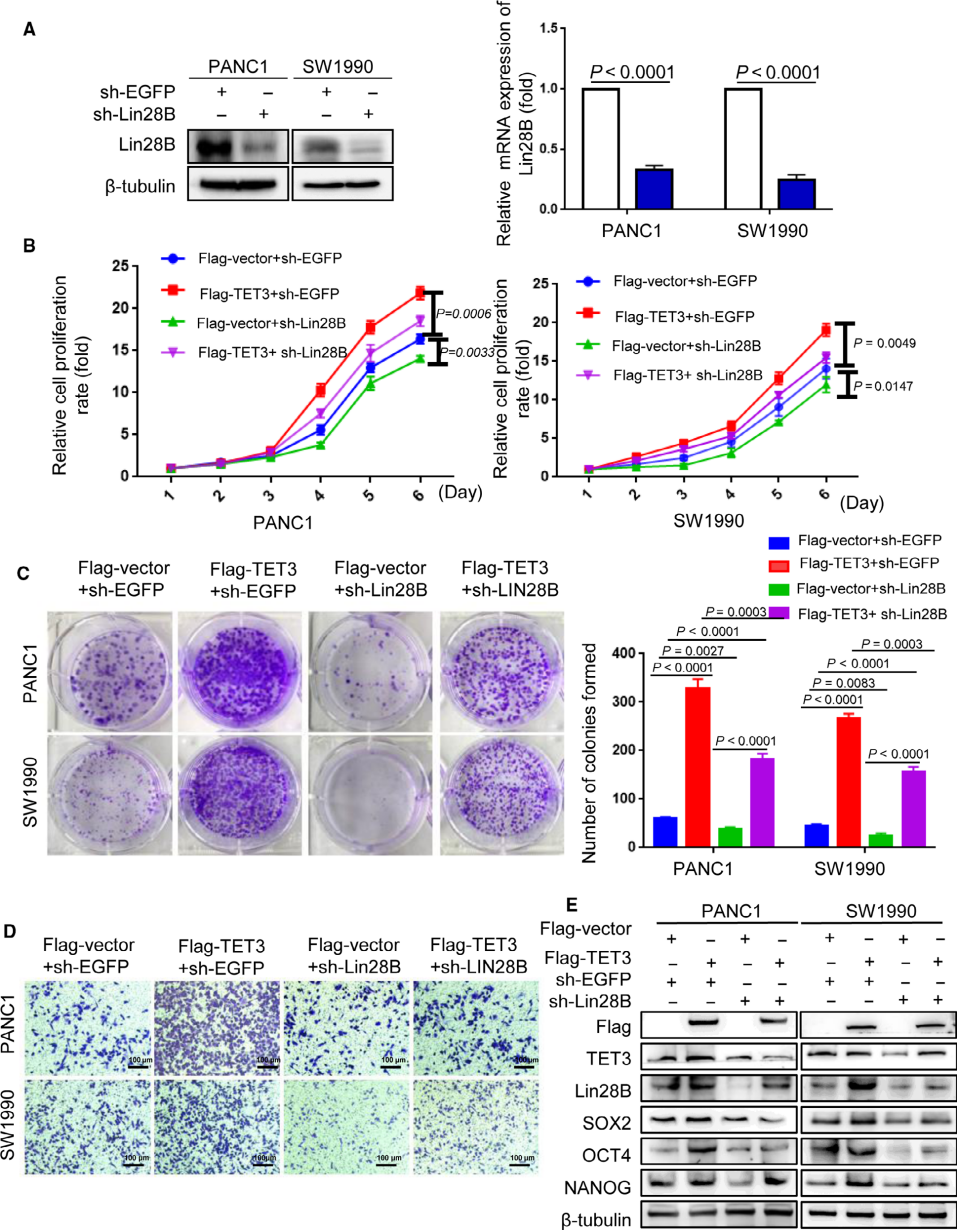
TET3 can rescue the effect of Lin28B knockdown in pancreatic cancer cells. (A) Expression of the plasmid sh‐Lin28B was analyzed in PANC1 and SW1990 cells, and sh‐EGFP was used to track the expression of sh‐Lin28B. (B–E) PANC1 and SW1990 cells were co‐transfected Flag‐vector and sh‐EGFP, Flag‐TET3 and sh‐EGFP, sh‐Lin28B and Flag‐vector, or Flag‐TET3 and sh‐Lin28B. (B) Cell proliferation, (C) colony formation capacity, (D) invasive ability and (E) the stem cell markers NANOG, OCT4 and SOX2 were analyzed in the two transfected cells by CCK‐8 assay, the colony formation assay, Transwell assay and Western blotting analysis, respectively. All experiments were repeated three times. Each error bar represents the standard error of the mean. Statistical analysis was calculated using Student’s *t*‐test or one‐way ANOVA test.

**Fig. 8:**
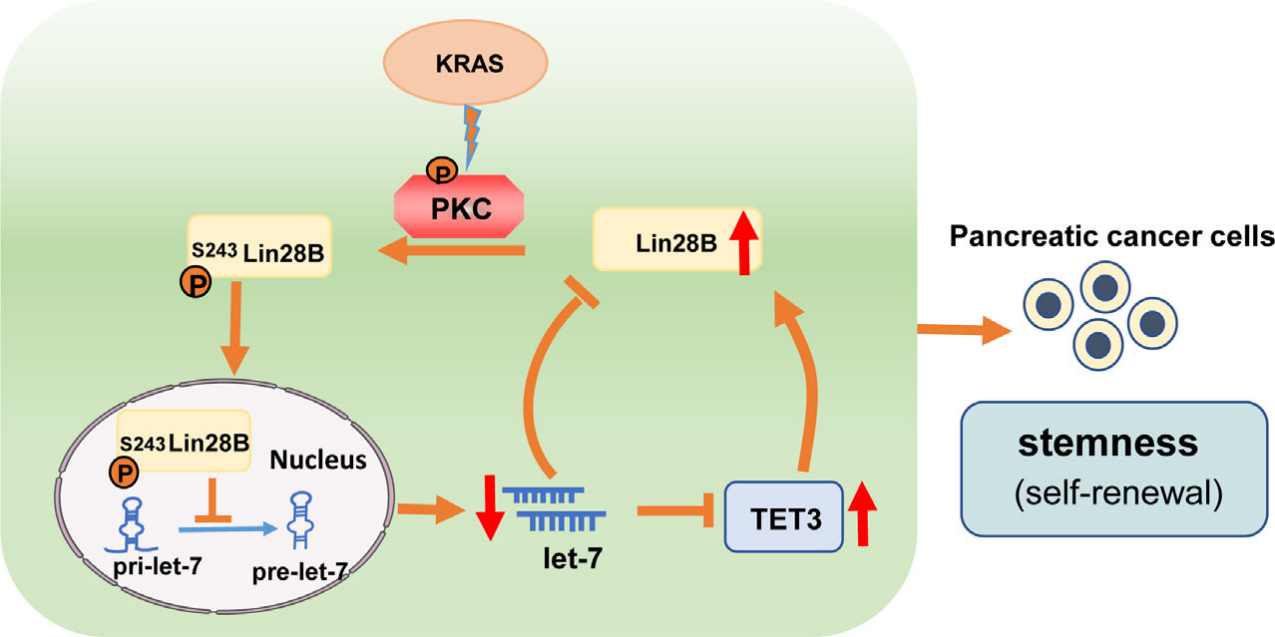
Scheme of PKCβ driven nuclear localization of Lin28B connected to a TET3‐mediated positive feedback loop. KRAS promotes nuclear localization of Lin28B by PKCβ, which directly phosphorylates Lin28B. Nuclear Lin28B indirectly elevates TET3 expression by repressing the bioactivity of let‐7i, and increased TET3 can feed back to promote Lin28B expression, which maintains the stemness of PC cells.

We further constructed sh‐Lin28B and sh‐EGFP plasmids. The protein and mRNA expression levels of Lin28B were significantly reduced in the sh‐Lin28B group compared with the sh‐EGFP group (Fig. 7A). In contrast, TET3 overexpressed in Lin28B knockdown cells resulted in a substantially increased proliferative capacity of PC cells (Fig. 7B,C). The decreased invasion of PC cells resulting from induced Lin28B knockdown was substantially rescued by TET3 overexpression in both PANC1 and SW1990 (Fig. 7D). In addition, restoration of TET3 strongly increased the expression of the stemness markers OCT4, NANOG and SOX2, which were suppressed by sh‐Lin28B (Fig. 7E). These results indicated that Lin28B promoted proliferation, invasion and stemness maintenance of PC cells by upregulating TET3, and that gain of TET3 expression can further feed back to promote Lin28B expression (Fig. 8).

## 4 Discussion

In the present study, we demonstrated that Lin28B is significantly elevated in PC tissues and enhanced the migration and proliferation of PC cells. Specifically, we found that KRAS promotes nuclear translocation of Lin28B by PKCβ, which directly binds to and phosphorylates Lin28B. Importantly, Lin28B can post‐transcriptionally increase TET3 expression through suppression of the miRNA level of let‐7i, and TET3 also significantly promoted Lin28B expression in a feedback mechanism. Thus, our results suggest that KRAS/Lin28B drives the let‐7/TET3 pathway to maintain the stemness of PC cells.

Lin28B is a highly conserved RNA‐binding and miRNA‐regulated protein involved in numerous biological processes, such as cell development, pluripotency, reprogramming and oncogenesis [29, 30]. However, the molecular mechanism of Lin28B nuclear translocation and its roles in the development and progression of pancreatic cancer remain poorly understood. Here, we found that PKCβ, as a binding partner of Lin28B, promotes Lin28B nuclear translocation and plays an important role in the KRAS‐mediated Lin28B regulatory mechanism. The PKC family consists of a number of serine‐threonine kinases which regulate a variety of cellular biological process and play a role in the carcinogenesis and maintenance of malignant phenotype. Individual PKC isozymes often play a distinct role in the regulation of cellular function through different subcellular localization, substrate selection and cofactor requirements [18]. In addition, KRAS facilitates sustained activation status for PKC, and PKC in turn affects KRAS activity by phosphorylating KRAS at S181 [31, 32]. In this study, bioinformatics analysis predicted the functional motifs between Lin28B and PKCβ. We further provide evidence that PKCβ interacts with Lin28B and S243 is critical to Lin28B–PKCβ binding, which promotes Lin28B nuclear localization. In addition, KRAS knockdown reduces the level of nuclear Lin28B through PKCβ. These results might explain the exact molecular mechanism of Lin28B nuclear translocation.

Previous studies have revealed that nuclear Lin28B blocks let‐7 processing in nucleus by sequestering primary let‐7 transcripts away from the Microprocessor [13]. The decrease of let‐7 blocked by Lin28 plays critical roles in CSC maintenance by increasing the expression of key oncogenes, such as KRAS, c‐Myc and HMGA2 [15, 33, 34]. Upregulated KRAS promotes Lin28B nuclear translocation by PKCβ, but nuclear Lin28B further represses the production of let‐7, forming a KRAS/Lin28B/let‐7 loop in pancreatic cancer. It is suggested that Lin28B plays a key role in KRAS‐driven pancreatic cancer.

TET3 can catalyse the conversion of 5‐methylcytosine to 5‐hydroxymethylcytosine, which can lead to DNA demethylation. TET3 has a critical role in the maintenance of PCSC by maintaining hypomethylation levels of pluripotency markers such as OCT4 and NANOG [24, 35, 36]. To further investigate the mechanism of Lin28B involved in stemness maintenance, we first examined the correlation between the expression level of Lin28B and TET3. Lin28B level was correlated with TET3 expression in PC tissues and cell lines. Furthermore, we demonstrated that overexpressed Lin28B increases TET3 expression by repressing let‐7i, which promotes the proliferation, invasion and stemness maintenance of PC cells. In consequence, TET3 functions as a novel downstream regulator of Lin28B and promotes the expression of Lin28B in a feedback mechanism.

## 5 Conclusions

This study elucidates the distinct mechanism of Lin28B nuclear translocation, and illustrates a new pathway of KRAS‐driven pancreatic cancer. Mechanistically, we provide the first evidence that KRAS promotes the nuclear translocation of Lin28B through PKCβ, and that nuclear Lin28B can indirectly elevate TET3 expression by inhibiting let‐7i. Intriguingly, upregulated TET3 can also feed back to rescue the Lin28B loss‐mediated suppression of stemness. Targeting this newly identified regulatory circuitry may provide a novel therapeutic strategy for pancreatic cancer.

## Conflict of interest

The authors declare no conflict of interest.

## Author contributions

YWL, DWW, MZ, HC, HZW, JYM, JXC, SHW and XFN carried out the experiments and analyzed the data. YLZ contributed many helpful suggestions about data processing and manuscript. AHG and MX contributed to the study design, manuscript drafting and provided funding for this study. YWL wrote the first and final draft of the manuscript. All authors read and approved the final manuscript.

### Peer Review

The peer review history for this article is available at https://publons.com/publon/10.1002/1878‐0261.12836.

## Supporting information


**Fig. S1.** (A) The relative expressed intensity of PKCβ protein in IP group (Fig. 2C). (B) The relative expressed intensity of Flag protein in nucleus (Fig. 2E). (C) Quantification of the average Lin28B nuclear/cytoplasmic fluorescence ratio per cell in Fig. 2F. (D) The relative expressed intensity of PKCβ in cytosol and nucleus (Fig. 3C). (E) Quantification of the average Lin28B nuclear/cytoplasmic fluorescence ratio per cell (Fig. 3D). Each error bar represents the standard error of the mean. Statistical analysis was calculated using Student’s *t*‐test.

**Fig. S2.** (A) The relative expressed intensity of PKCβ in cytosol and nucleus (Fig. 3E). (B) Quantification of the average Lin28B nuclear/cytoplasmic fluorescence ratio per cell in Fig. 3F. (C) KRAS protein levels were reduced in PANC1 cells transfected with sh‐Lin28B. (D) TET3 knockdown downregulated the expression of KRAS protein in PANC1 cells. (E) The expression of Lin28B was correlated with the levels of TET3 and KRAS in human pancreatic carcinoma. All experiments were repeated three times. Each error bar represents the standard error of the mean. Statistical analysis was calculated using Student’s *t*‐test.
