# Supplementary figures and images for: The KRAS/Lin28B axis maintains stemness of pancreatic cancer cells via the let‐7i/TET3 pathway
Source: Mol Oncol. 2020 Nov 28;15(1):262–78. doi: 10.1002/1878-0261.12836 (PMC7782082; doi:10.1002/1878-0261.12836)

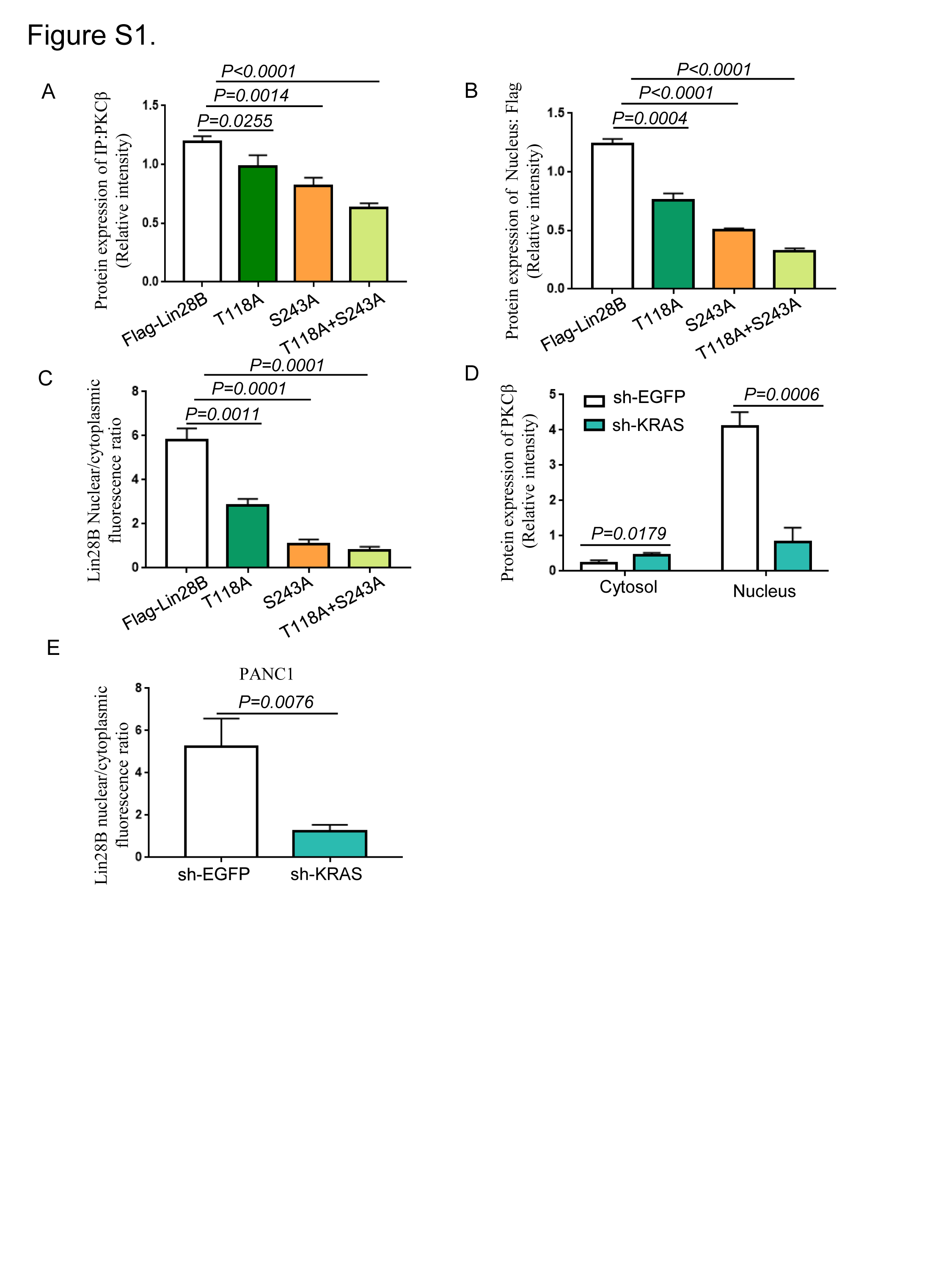

Supplement: Supplementary file 1 — Fig. S1. (A) The relative expressed intensity of PKCβ protein in IP group (Fig. 2C). (B) The relative expressed intensity of Flag protein in nucleus (Fig. 2E). (C) Quantification of the average Lin28B nuclear/cytoplasmic fluorescence ratio per cell in Fig. 2F. (D) The relative expressed intensity of PKCβ in cytosol and nucleus (Fig. 3C). (E) Quantification of the average Lin28B nuclear/cytoplasmic fluorescence ratio per cell (Fig. 3D). Each error bar represents the standard error of the mean. Statistical analysis was calculated using Student’s t‐test. [file MOL2-15-262-s001.tif]

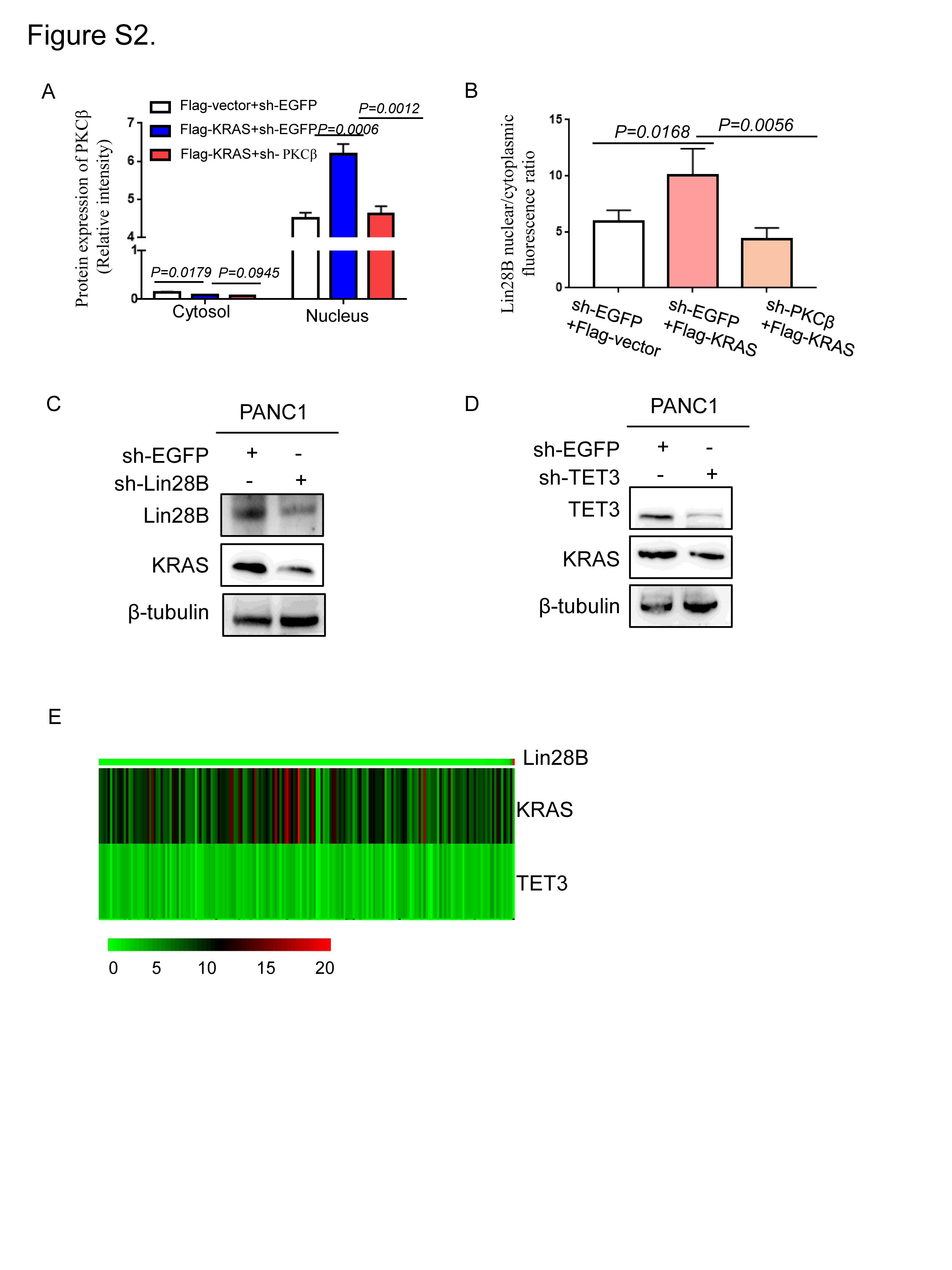

Supplement: Supplementary file 2 — Fig. S2. (A) The relative expressed intensity of PKCβ in cytosol and nucleus (Fig. 3E). (B) Quantification of the average Lin28B nuclear/cytoplasmic fluorescence ratio per cell in Fig. 3F. (C) KRAS protein levels were reduced in PANC1 cells transfected with sh‐Lin28B. (D) TET3 knockdown downregulated the expression of KRAS protein in PANC1 cells. (E) The expression of Lin28B was correlated with the levels of TET3 and KRAS in human pancreatic carcinoma. All experiments were repeated three times. Each error bar represents the standard error of the mean. Statistical analysis was calculated using Student’s t‐test. [file MOL2-15-262-s002.tif]
